# Electronic structures and topological properties in nickelates *Ln*_*n*+1_Ni_*n*_O_2*n*+2_

**DOI:** 10.1093/nsr/nwaa218

**Published:** 2020-09-02

**Authors:** Jiacheng Gao, Shiyu Peng, Zhijun Wang, Chen Fang, Hongming Weng

**Affiliations:** Beijing National Laboratory for Condensed Matter Physics, Institute of Physics, Chinese Academy of Sciences, Beijing 100190, China; School of Physical Sciences, University of Chinese Academy of Sciences, Beijing 100190, China; Beijing National Laboratory for Condensed Matter Physics, Institute of Physics, Chinese Academy of Sciences, Beijing 100190, China; School of Physical Sciences, University of Chinese Academy of Sciences, Beijing 100190, China; Beijing National Laboratory for Condensed Matter Physics, Institute of Physics, Chinese Academy of Sciences, Beijing 100190, China; School of Physical Sciences, University of Chinese Academy of Sciences, Beijing 100190, China; Beijing National Laboratory for Condensed Matter Physics, Institute of Physics, Chinese Academy of Sciences, Beijing 100190, China; Songshan Lake Materials Laboratory, Dongguan 523808, China; Kavli Institute for Theoretical Sciences, Chinese Academy of Sciences, Beijing 100190, China; Beijing National Laboratory for Condensed Matter Physics, Institute of Physics, Chinese Academy of Sciences, Beijing 100190, China; School of Physical Sciences, University of Chinese Academy of Sciences, Beijing 100190, China; Songshan Lake Materials Laboratory, Dongguan 523808, China; Physical Science Laboratory, Huairou National Comprehensive Science Center, Beijing 101407, China

**Keywords:** nickelate superconductors, band representations, topological Dirac points, DFT+Gutzwiller calculations

## Abstract

After the significant discovery of the hole-doped nickelate compound Nd_0.8_Sr_0.2_NiO_2_, analyses of the electronic structure, orbital components, Fermi surfaces and band topology could be helpful to understand the mechanism of its superconductivity. Based on first-principle calculations, we find that Ni $3d_{x^2-y^2}$ states contribute the largest Fermi surface. The $Ln 5d_{3z^2-r^2}$ states form an electron pocket at Γ, while 5*d*_*xy*_ states form a relatively bigger electron pocket at A. These Fermi surfaces and symmetry characteristics can be reproduced by our two-band model, which consists of two elementary band representations: *B*_1*g*_@1*a* ⊕ *A*_1*g*_@1*b*. We find that there is a band inversion near A, giving rise to a pair of Dirac points along M-A below the Fermi level upon including spin-orbit coupling. Furthermore, we perform density functional theory based Gutzwiller (DFT+Gutzwiller) calculations to treat the strong correlation effect of Ni 3d orbitals. In particular, the bandwidth of $3d_{x^2-y^2}$ has been renormalized largely. After the renormalization of the correlated bands, the Ni 3*d*_*xy*_ states and the Dirac points become very close to the Fermi level. Thus, a hole pocket at A could be introduced by hole doping, which may be related to the observed sign change of the Hall coefficient. By introducing an additional Ni 3*d*_*xy*_ orbital, the hole-pocket band and the band inversion can be captured in our modified model. Besides, the nontrivial band topology in the ferromagnetic two-layer compound La_3_Ni_2_O_6_ is discussed and the band inversion is associated with Ni $3d_{x^2-y^2}$ and La 5*d*_*xy*_ orbitals.

## INTRODUCTION

After the discovery of high-T_*c*_ superconductivity in the cuprates [1,2], mixed-valent nicklates with similar crystals and electronic configurations as cuprates have attracted a lot of attention  [3]. In particular, the configuration of Ni^+^ in infinite-layer nickelates *Ln*NiO_2_ (*Ln* = La, Nd, Pr) is almost identical to that of Cu^++^ in the parent compounds of cuprates. Although much effort has been devoted along this direction in the past two decades, the possible superconductivity in mixed-valent nicklates remains elusive. Until very recently, the superconductivity with T_*c*_ = 9 ∼ 15 K was discovered in hole doped Nd_0.8_Sr_0.2_NiO_2_ for the first time [4]. For the parent compound NdNiO_2_, previous studies have established several experimental facts that are distinct from the parent compound of cuprates. First, no long-range magnetic order is observed experimentally [5,6], while an antiferromagnetically ordered state is formed in the cuprates [7]. Second, NdNiO_2_ exhibits a metallic behavior above 50 K [4], while the parent cuprates are Mott insulators [8]. Third, the superconductivity (so far) is only found in the hole doped Nd_0.8_Sr_0.2_NiO_2_, while it is found in the electron-doped cuprate Sr_1−*x*_La_*x*_CuO_2_ in the same structure [9]. These experimental facts indicate that the ground state of the parent nickelates could have significant difference from the cuprates. Analyses of their electronic band structures, orbital components, Fermi surfaces and the band topology are needed. In addition, a minimal-band effective model is very helpful to further understand the mechanism of superconductivity.

In this work, we perform detailed first-principle calculations within the framework of density functional theory (DFT). The obtained band structure of the parent (undoped) compound NdNiO_2_ is similar to that of LaNiO_2_ reported previously [10] and PrNiO_2_ (Nd-4*f* and Pr-4*f* orbitals are treated as core states). In this article, LaNiO_2_ is taken as a representative and the band structures of NdNiO_2_ and PrNiO_2_ are presented in the online supplementary material. In the undoped case, there are three bands intersecting the Fermi level (*E*_*F*_), mainly from Ni $3d_{x^2-y^2}$, La 5*d*_*xy*_ and La $5d_{3z^2-r^2}$ orbitals. They form a large cylinderlike electron pocket (EP) surrounding the Γ-Z line, a spherelike EP at A and a comparatively smaller spherelike EP at Γ, respectively. These Fermi surfaces and symmetry characteristics can be reproduced by our two-band model, which consists of two elementary band representations (EBRs): *B*_1*g*_@1*a* ⊕ *A*_1*g*_@1*b*. The EBR of *B*_1*g*_@1*a* refers to the $3d_{x^2-y^2}$ orbital at Wyckoff site 1*a*[0,0,0], while the EBR of *A*_1*g*_@1*b* refers to the *A*_1*g*_ orbital at Wyckoff site 1*b*[0,0,0.5], where no atoms sit.

We find that a band inversion near A happens between the Ni 3*d*_*xy*_ states and *Ln* 5*d*_*xy*_ states (the effect of Coulomb interaction *U* is discussed in the online supplementary material). With small *U* and spin-orbital coupling (SOC), this gives rise to a pair of Dirac points along M-A. After considering the renormalization of Ni 3*d* bands, the Dirac point becomes very close to the charge neutrality level and accessible by hole doping. As a result, a hole pocket (HP) could emerge at A, which may be responsible for the sign change of the Hall coefficient in the experiment  [4]. By introducing an additional Ni 3*d*_*xy*_ orbital, the hole-pocket band and the band inversion can be captured in the modified model. Besides, the nontrivial band topology in the ferromagnetic two-layer compound La_3_Ni_2_O_6_ (*n*=2) is discussed and a band inversion happens between the Ni $3d_{x^2-y^2}$ and La 5*d*_*xy*_ orbitals.

## CRYSTAL STRUCTURE AND METHODOLOGY

**Figure 1.**
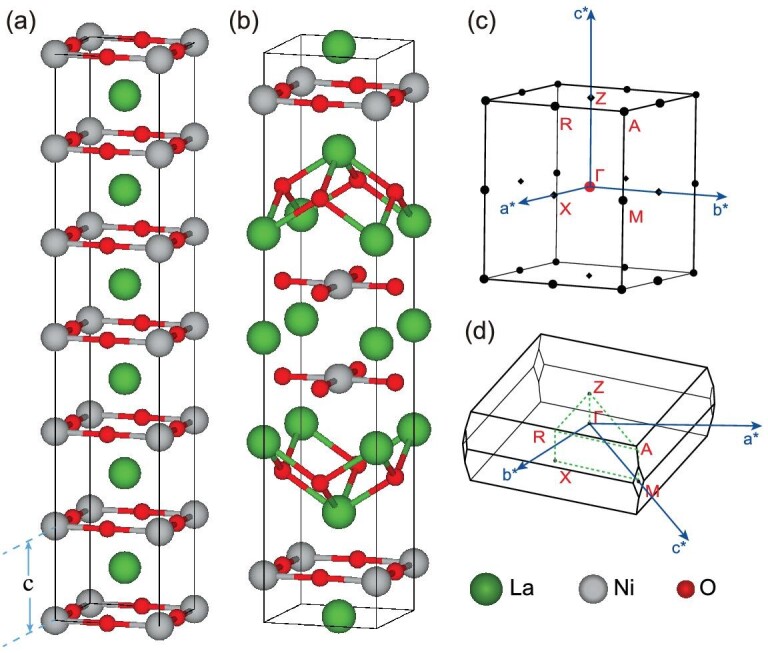
Crystal structures and Brillouin zones. The crystals of the two end members LaNiO_2_ (*n* = ∞, *P*4/*mmm*) and La_3_Ni_2_O_6_ (*n* = 2, *I*4/*mmm*) are presented in (a) and (b), respectively. The panel (a) contains six unit cells of LaNiO_2_ (*c* is the lattice parameter in the $z$ direction). The primitive reciprocal lattice vectors and high-symmetry *k* points are indicated in the first Brillouin zones of (c) LaNiO_2_ and (d) La_3_Ni_2_O_6_. a^*^, b^*^ and c^*^ refer to the primitive reciprocal lattice vectors.

The parent compound LaNiO_2_ can be obtained from the perovskites LaNiO_3_ by removing the apical oxygens (i.e. *b* site), as shown in Fig. 1(a). Consequently, it has a tetragonal lattice, and has the same planes as the cuprate superconductors with Ni^+^ instead of Cu^++^ ions. Similarly, the two-layer nickelate La_3_Ni_2_O_6_ (see Fig. 1(b)) can be produced [11] from the two-layer perovskite La_3_Ni_2_O_7_. We perform first-principle calculations using the VASP package [12,13] based on density functional theory with the projector augmented wave method [14,15]. The generalized gradient approximation (GGA) and the exchange-correlation functional of Perdew, Burke and Ernzerhof [16] are employed. The kinetic energy cutoff is set to 500 eV for the plane wave basis. A 10 × 10 × 10 *k* mesh for self-consistent Brillouin zone (BZ) sampling is adopted. The experimental lattice parameters of LaNiO_2_ and La_3_Ni_2_O_6_ are employed  [4,11].

## RESULTS AND DISCUSSIONS

### Band structure and density of states

**Figure 2.**
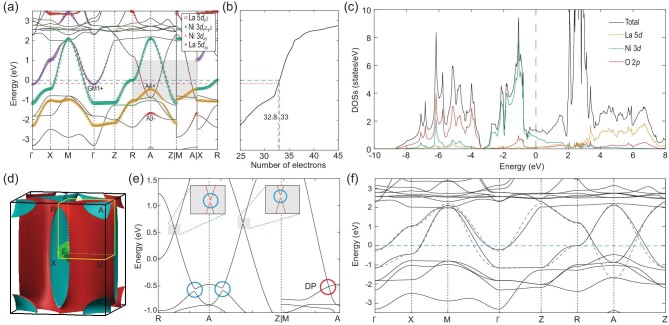
(a) The band structure of LaNiO_2_ without SOC. The weights of the La $5d_{z^2}$, Ni $3d_{x^2-y^2}$, Ni 3*d*_*xy*_ and La 5*d*_*xy*_ states are indicated by the size of the purple squares, green circles, yellow triangles and red diamonds, respectively. The La $5d_{z^2}$ band at Γ is labeled GM1+, while the Ni 3*d*_*xy*_ and La 5*d*_*xy*_ bands at A are labeled A4+ and A3−, respectively. The blue dashed line represents the Fermi level *E*_*F*_ and the red dashed line represents the estimated chemical potential in the hole-doped Nd_0.8_Sr_0.2_NiO_2_. The total number of electrons as a function of the chemical potential is plotted in (b). The partial density of states (DOSs) is given in (c). The Fermi surfaces of LaNiO_2_ are shown in (d). In the band structure with SOC (e), the crossings in the shadowed area of (a) are gapped except the Dirac point (DP) along M-A. The bands of our two-band model are shown as blue dashed lines in (f).

We first perform first-principle calculations on LaNiO_2_ without SOC. The band structure is presented in Fig. 2(a) and the total density of states is given in Fig. 2(c). The blue dashed horizontal line corresponds to the charge neutrality level of the undoped case. The red dashed line in Fig. 2(b) is the theoretically estimated chemical potential for the 20% hole-doped superconductivity Nd_0.8_Sr_0.2_NiO_2_. Moreover, the partial DOSs are also computed for O 2*p*, Ni 3*d* and La 5*d* orbitals, respectively. Since the main quantum numbers of different atom’s orbitals are distinct, we call them 2*p*, 3*d* and 5*d* orbitals (states) for short in the following discussion. From the plotted partial DOSs in Fig. 2(c), we note that 2*p* states are mainly located from −10 to −3.5 eV below *E*_*F*_, while 3*d* states are around *E*_*F*_, from −3.5 to 1.5 eV. The situation is much different from the situation in copper-based superconductors, where O 2*p* states are slightly below *E*_*F*_ and hybridize strongly with Cu 3*d* states  [10]. In addition, from the orbital projections in Fig. 2(a), we note that there are $5d_{3z^2-r^2}$ states at Γ and 5*d*_*xy*_ states at *A* around *E*_*F*_, suggesting that the 5*d* states are more extended, compared to the Ca 3*d* states in CaCuO_2_  [17].

### Evolution of Fermi surfaces

At the charge neutrality level, we find that there are three bands crossing *E*_*F*_, which are mainly from $3d_{x^2-y^2}$, $5d_{z^2}$ and 5*d*_*xy*_ orbitals, respectively. The weights of these orbitals are depicted by the size of the different symbols in Fig. 2(a). Therefore, as shown in Fig. 2(d), three EPs are formed: (i) the $3d_{x^2-y^2}$ orbital forms the largest electron pocket around the Γ and *Z* points, which has a strong two-dimensional (2D) feature; (ii) the second larger EP is nearly a sphere around A, formed by the 5*d*_*xy*_ orbital; (iii) the smallest EP is a sphere around Γ, formed by the $5d_{3z^2-r^2}$ orbital (which is also hybridized with the Ni $3d_{3z^2-r^2}$ orbital in the DFT calculations, yet we still call it $5d_{3z^2-r^2}$ for simplicity).

In the hole-doped superconductor Nd_0.8_Sr_0.2_NiO_2_, the estimated chemical potential of the 20% Sr-doped level is denoted by a red dashed line, which corresponds to 32.8 electrons per unit cell (the charge neutrality level corresponds to 33 electrons). Needless to say that all the electron pockets become smaller with hole doping. In particular, the $5d_{3z^2-r^2}$-orbital-formed Γ-centered EP is about to be removed. On the other hand, the states from the 3*d*_*xy*_ orbital become closer to the chemical potential, especially in the vicinity of point A.

### Band inversion and Dirac points

The band crossings along R-A, A-Z and M-A are protected by *m*_001_, *m*_110_ and C_4$z$_, respectively. After considering SOC, the band crossings open small gaps (blue circles) along the mirror protected R-A and A-Z lines, but remain gapless along the C_4$z$_-invariant line M-A, as shown in Fig. 2(e). The gapless Dirac points along M-A [highlighted in the red circle in Fig. 2(e)] are protected by *C*_4$v$_ symmetry. Namely, the two doubly degenerate bands belong to different 2D irreducible representations (IRs) of the *C*_4$v$_ double group. In our GGA+U calculations, we find that the band inversion is sensitive to the value of the Coulomb interaction *U*, as we show in the online supplementary material.

### Analysis of EBRs and orbitals

By doing an analysis of EBRs in the theory of topological quantum chemistry [18], we can also obtain orbital information in real space. An EBR of ρ@*q* is labeled by the Wyckoff position *q* and the IR ρ of its site symmetry group. The IRs of the six low-energy bands at maximal high symmetry *k* points  [19,20] are computed without SOC. The results are listed in Table 1. In the crystal of LaNiO_2_, the Ni atom sits at Wyckoff position 1*a*[0, 0, 0], while the La atom sits at Wyckoff position 1*d*[0.5, 0.5, 0.5]. Both Wyckoff positions have the site-symmetry group of 4/*mmm*. Note that five *d* orbitals only support the IRs of $A_{1g}(d_{3z^2-r^2})$, $B_{1g}(d_{x^2-y^2})$, *B*_2*g*_ (*d*_*xy*_) and *E*_*g*_ (*d*_*xz*, *yz*_) under the single group of 4/*mmm*.

**Table 1.** The upper rows give the IRs for the lowest six bands in Fig. 2(a) at the maximal high-symmetry points in SG 123. The IRs are given in ascending energy order. The notation *Zm*(*n*) denotes the IR *m* at the *Z* point with degeneracy *n*. The lower rows give the elementary band representations, labeled as ρ@*q*. Here, ρ indicates the IR supported by the orbital(s), while *q* stands for the Wyckoff site, where the orbital(s) is (are) located. See https://www.cryst.ehu.es/cgi-bin/cryst/programs/bandrep.pl [21–23] for all eBRs and IRs.The gray IRs indicate that those energy bands are at least 1.0 eV above *E*_*F*_.

	A	Γ	M	Z	R	X
DFT bands	A3 −(1)	GM1+(1)	M1+(1)	Z4+(1)	R4+(1)	X1+(1)
	A1+(1)	GM4+(1)	M4+(1)	Z5+(2)	R1+(1)	X4+(1)
	A5+(2)	GM5+(2)	M5+(2)		R2+(1)	X2+(1)
				Z1+(1)	R3+(1)	X3+(1)
	A4 +(1)	GM2+(1)		Z2+(1)	R1+(1)	X1+(1)
	A2+(1)	GM1+(1)	M5 −(2)	Z1+(1)	R2 −(1)	X4 −(1)
EBRs						
*A*_1*g*_@1*a*	A1+	GM1+	M1+	Z1+	R1+	X1+
*B*_1*g*_@1*a*	A2+	GM2+	M2+	Z2+	R1+	X1+
*B*_2*g*_@1*a*	A4+	GM4+	M4+	Z4+	R2+	X2+
*E*_*g*_@1*a*	A5+	GM5+	M5+	Z5+	R3+	X3+
					R4+	X4+
*A*_1*g*_@1*d*	A2 −	GM1+	M4+	Z3 −	R3+	X4 −
*B*_2*g*_@1*d*	A3 −	GM4+	M1+	Z2 −	R4+	X3 −
*A*_1*g*_@1*b*	A3 −	GM1+	M1+	Z3 −	R2 −	X1+

The results of the EBR analysis on atomic orbitals are as follows. First, by switching the IRs of A3− and A4+ at A, we find that the four occupied bands can be represented as the sum of three EBRs: *A*_1*g*_@1*a* ⊕ *B*_2*g*_@1*a* ⊕ *E*_*g*_@1*a*. Among them, the 3*d*_*xy*_-based EBR *B*_2*g*_@1*a* has highest states at A, which may intersect with the chemical potential in the hole-doping case. Second, the band of the $3d_{x^2-y^2}$-induced EBR *B*_1*g*_@1*a* is clearly shown by the weights in Fig. 2(a). There is no doubt that the $3d_{x^2-y^2}$ orbital contributes the largest Fermi surface. Third, the IR GM1+ at Γ is from the $5d_{3z^2-r^2}$-induced EBR *A*_1*g*_@1*d*. Last, the inverted IR A3− is from the 5*d*_*xy*_-induced EBR *B*_2*g*_@1*d*. So far, all of the orbital compounds are consistent with our DFT calculations in Fig. 2(a).

We now aim to construct a minimal effective model to reproduce the bands and symmetry characteristics near *E*_*F*_. Besides the $3d_{x^2-y^2}$-induced EBR of *B*_1*g*_@1*a*, the two IRs of A3− and GM1+ can be generated in the EBR of *A*_1*g*_@1*b*[0,0,0.5]. Therefore, we derive a two-band model, consisting of two EBRs: *B*_1*g*_@1*a* ⊕ *A*_1*g*_@1*b*, which reproduces the exact IRs of the bands near *E*_*F*_ from the DFT calculations. Even through there is no actual atomic orbital at Wyckoff position 1*b* (the apical oxygens are removed), it could be formed by the hybridization of the atomic orbitals on other sites. The two-band model will be constructed for the undoped case in the following section.

### Two-band effective model

Under the basis of the *B*_1*g*_ orbital at the 1*a* Wyckoff position and the *A*_1*g*_ orbital at the 1*b* Wyckoff position, the tight binding model is constructed as follows. The diagonal terms in the Hamiltonian are

(1) \begin{eqnarray*} T_{\alpha \alpha } &=& t_{\alpha \alpha }^{(0,0,0)} + 2t_{\alpha \alpha }^{(1,0,0)}[\cos (k_x)+\cos (k_y)] \nonumber \\ &&+\,\, 2t_{\alpha \alpha }^{(0,0,1)}\cos (k_z)\nonumber\\ &&+\,\, 4t_{\alpha \alpha }^{(1,1,0)}\cos (k_x)\cos (k_y) \nonumber \\ &&+\,\, 4t_{\alpha \alpha }^{(1,0,1)}\cos (k_z)[\cos (k_x)+\cos (k_y)] \nonumber \\ &&+\,\, 8t_{\alpha \alpha }^{(1,1,1)}\cos (k_x)\cos (k_y)\cos (k_z), \end{eqnarray*}

where α = 1, 2 represent the *B*_1*g*_ orbital and the *A*_1*g*_ orbital, respectively. Here $t_{\beta \alpha }^{(l,m,n)}$ stands for the hopping parameter from orbital β of the original cell to α of the (*l*, *m*, *n*) cell:

(2) \begin{equation*} t_{\beta \alpha }^{(l,m,n)} \equiv \langle \beta ;000 | \hat{H} | \alpha ;lmn \rangle. \end{equation*}

In the off-diagonal term, the nearest and next-nearest hoppings between different orbitals are given as

(3) \begin{equation*} S = t_{21}^{(1,0,0)} (1+e^{ik_z})[4\cos (k_x) - 4\cos (k_y)]. \end{equation*}

Thus, our two-band model Hamiltonian is written as

(4) \begin{equation*} H_2(k) = \left(\begin{array}{cc}T_{11} &\quad \dagger \\ S &\quad T_{22} \\ \end{array}\right), \end{equation*}

where the dagger symbol means the Hermitian conjugate of the lower left part of the Hamiltonian matrix (same below). The fitting results are shown as blue dashed lines in Fig. 2(f) and the parameters can be found in Box 1. This two-band model can reproduce all the Fermi surfaces and symmetry characteristics obtained from the DFT calculations. It can be used for further study of superconductivity in the systems.

**Box 1.** The hopping parameters for the two-band and three-band model Hamiltonians.

Parameters for the two-band model							
	$t_{11}^{(0,0,0)}$	0.1470	$t_{22}^{(0,0,0)}$	0.8318	$t_{21}^{(1,0,0)}$	0.0098	
	$t_{11}^{(1,0,0)}$	−0.4125	$t_{22}^{(1,0,0)}$	0.0913			
	$t_{11}^{(0,0,1)}$	−0.0538	$t_{22}^{(0,0,1)}$	0.0650			
	$t_{11}^{(1,1,0)}$	0.0894	$t_{22}^{(1,1,0)}$	−0.0606			
	$t_{11}^{(1,0,1)}$	0.0000	$t_{22}^{(1,0,1)}$	0.1988			
	$t_{11}^{(1,1,1)}$	0.0134	$t_{22}^{(1,1,1)}$	0.0281			
Parameters for the three-band model							
$t_{11}^{(0,0,0)}$	0.0100	$t_{22}^{(0,0,0)}$	0.8280	$t_{33}^{(0,0,0)}$	−0.7960	$t_{21}^{(1,0,0)}$	0.0098
$t_{11}^{(1,0,0)}$	0.0042	$t_{22}^{(1,0,0)}$	−0.0510	$t_{33}^{(1,0,0)}$	−0.1655	$t_{31}^{(1,0,0)}$	−0.0132
$t_{11}^{(0,0,1)}$	0.0378	$t_{22}^{(0,0,1)}$	−0.0079	$t_{33}^{(0,0,1)}$	−0.0360	$t_{32}^{(1,1,1)}$	0.0001
$t_{11}^{(1,1,0)}$	0.0043	$t_{22}^{(1,1,0)}$	0.0105	$t_{33}^{(1,1,0)}$	−0.0497		
$t_{11}^{(1,0,1)}$	−0.1338	$t_{22}^{(1,0,1)}$	0.0000	$t_{33}^{(1,0,1)}$	0.0105		
$t_{11}^{(1,1,1)}$	0.0038	$t_{22}^{(1,1,1)}$	0.0018	$t_{33}^{(1,1,1)}$	−0.0113		

### DFT+Gutzwiller method

To treat the correlation effect of five Ni 3*d* orbitals, we employ the DFT+Gutzwiller method  [24,25]. The corresponding Gutzwiller trial wave function has been constructed as $\left|G\right\rangle = \hat{P}\left|0\right\rangle$ with

(5) \begin{equation*} \hat{P} = \prod _i \hat{P}_i = \prod _i \sum _{\Gamma \Gamma ^{\prime }} \lambda _{i;\Gamma \Gamma ^{\prime }} \left|i,\Gamma \right\rangle \left\langle i,\Gamma ^{\prime }\right|, \end{equation*}

where |0〉 is the noninteracting wave function and $\hat{P}$ is the Gutzwiller local projector with |*i*, Γ〉 the atomic eigenvectors on site *i*. The $\lambda _{i;\Gamma \Gamma ^{\prime }}$ are the so-called Gutzwiller variational parameters that adjust the weights of different local atomic configurations. Ground states are obtained by minimizing the total energy *E* = 〈*G*|(*H*_*tb*_ + *H*_*dc*_ + *H*_*int*_)|*G*〉 with some Gutzwiller constraints. The noninteracting Hamiltonian (*H*_*tb*_) is extracted using the Wannier90 package  [26] from the DFT calculations without SOC, which contains two La $5d_{3z^2-r^2,xy}$ orbitals and five Ni 3*d* orbitals. The double-counting term *H*_*dc*_ is given self-consistently. The on-site interacting term takes the Slater–Kanamori rotationally invariant atomic interaction  [27]

(6) \begin{eqnarray*} H_{int}&=& U\sum _\alpha \hat{n}_{a\uparrow }\hat{n}_{a\downarrow }+\frac{U^{\prime }}{2}\sum _{a\ne b}\sum _{\sigma \sigma ^{\prime }} \hat{n}_{a\sigma }\hat{n}_{b\sigma ^{\prime }} \nonumber \\ &&-\,\frac{J}{2}\sum _{a\ne b}\sum _{\sigma } c^\dagger _{a\sigma }c_{a-\sigma }c^\dagger _{b-\sigma }c_{b\sigma } \nonumber \\ &&-\,\frac{J^{\prime }}{2}\sum _{a\ne b} c^\dagger _{a\uparrow }c^\dagger _{a\downarrow }c_{b\uparrow }c_{b\downarrow }, \end{eqnarray*}

where $c^\dagger _{a\sigma }(c_{a\sigma })$ creates (annihilates) an electron in the state of the orbital *a* and the spin σ, and $\hat{n}_{a\sigma }=c^\dagger _{a\sigma } c_{a\sigma }$.

**Figure 3.**
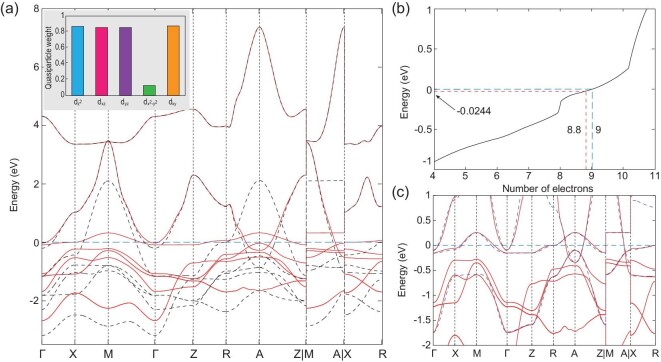
(a) The band structure of the noninteracting tight-binding Hamiltonian *H*_*tb*_, extracted by the Wannier90 package, is given by black dashed lines, while the DFT+Gutzwiller bands are plotted in red solid lines. The inset of (a) shows the quasiparticle weights of five 3*d* orbitals. In panel (b) we show the total number of electrons as a function of the chemical potential in the DFT+Gutzwiller band structure. In panel (c), the bands of the three-band model are given by blue dashed lines.

For simplicity, we adopt diagonal variational parameters, which mean that $\lambda _{i;\Gamma \Gamma ^{\prime }} = \lambda _{i;\Gamma \Gamma } \delta _{\Gamma \Gamma ^{\prime }}$ here. Five Ni 3*d* orbitals are treated as correlated orbitals in the calculations (which correspond to 10 bands upon considering the spin degree of freedom). We take a Coulomb interaction *U* of 5 eV, Hund’s coupling *J* of 0.18*U*, where *U*^′^ = *U* − 2*J* and *J*^′^ = *J*. Besides, the occupancy of Ni 3*d* orbitals has been forced to be 8.462 obtained from the DFT calculations. The results show that the quasiparticle weight of the $d_{x^2-y^2}$ orbital is very small, 0.12, while the weights of the other four orbitals are about 0.85. After considering the renormalization of the correlated 3*d* orbitals, the modified band structure is obtained and shown in Fig. 3(a). Two significant features are found after the Gutzwiller correction: (i) the bandwidth of $3d_{x^2-y^2}$ has been largely renormalized, leading to a DOS peak around *E*_*F*_, which may contribute to the large peak around zero energy in the resonant inelastic X-ray scattering spectrum (RIXS) [28]; (ii) the 3*d*_*xy*_ states near the A point become very close to *E*_*F*_. As a result, a HP could be induced by hole doping, which may be related to the observed sign change of the Hall coefficient.

In addition, the band inversion between the 3*d*_*xy*_ states and 5*d*_*xy*_ states near the A point could be important in the hole-doped nickelate compound Nd_0.8_Sr_0.2_NiO_2_, since they are very close to *E*_*F*_. Therefore, we modify our model by simply adding an additional 3*d*_*xy*_ orbital to capture the potential band inversion in this hole-doped compound. The modified model is written as

(7) \begin{equation*} H_3(k) = \left(\begin{array}{ccc}T_{11} &\quad &\quad \dagger \\ S &\quad T_{22} &\quad \\ W &\quad P &\quad T_{33} \\ \end{array}\right) \end{equation*}

with

$$\begin{eqnarray*}
P = 4t_{31}^{(1,0,0)}(1+e^{1k_z})[\cos (k_x)-\cos (k_y)] , \nonumber\\
W = 8t_{32}^{(1,1,1)}\cos (k_x)\cos (k_y)\cos (k_z).
\end{eqnarray*}$$

The parameters are obtained by fitting with the renormalized bands, as shown in Box 1. The results of the modified model *H*_3_(*k*) are shown as blue dashed lines in Fig. 3(c), which fit very well with the DFT+Gutzwiller bands, which can be compared with the angle-resolved photoemission spectroscopy (ARPES) experimental data. In any case, a four-band model constructed totally from the real atomic orbitals is presented in the online supplementary material.

### Ferromagnetic state in La_3_Ni_2_O_6_

**Figure 4.**
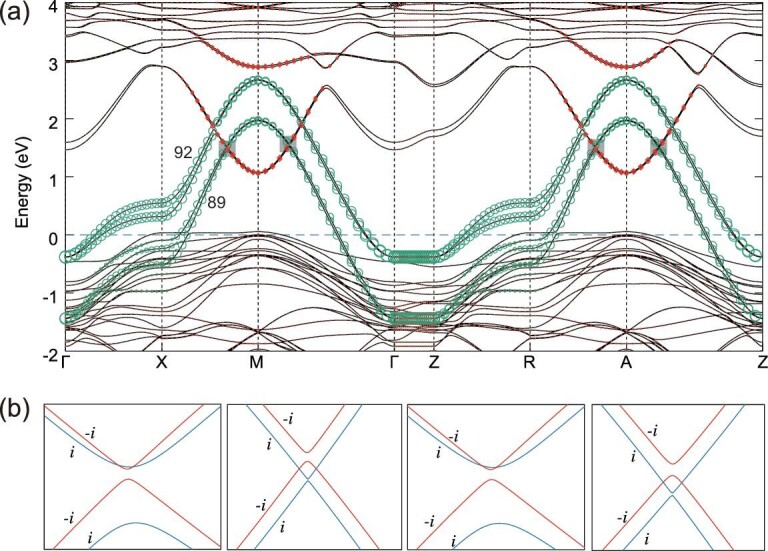
(a) The ferromagnetic band structure of the La_3_Ni_2_O_6_ with SOC. The weights of La 5*d*_*xy*_ and Ni $3d_{x^2-y^2}$ states are indicated by the size of red diamonds and green circles, respectively. (b) The crossing points between 90th and 91st bands. Bands with different mirror eigenvalues are shown as blue (for +*i*) and red (for −*i*) lines.

To better understand the electronic structures of *T*^′^-type La_*n*+1_Ni_*n*_O_2*n*+2_ compounds (*n* ≥ 2), we calculate the other end member La_3_Ni_2_O_6_ (i.e. *n* = 2 corresponds to the most reduced case) [29,30]. The ferromagnetic band structure of La_3_Ni_2_O_6_ with SOC is shown in Fig. 4(a). The $z$-oriented magnetism [31] reduces the symmetry from type-II magnetic SG 139 to type-I magnetic SG 87. Near the M/A point, the four downward parabolic bands (i.e. 89–92 bands) are mainly from the $3d_{x^2-y^2}$ states of Ni atoms in two planar Ni–O layers, while the two upward parabolic bands (i.e. 93–94 bands) are mainly from the 5*d*_*xy*_ states of the La atoms sandwiched by two Ni–O planes (Fig. 1(b)). Looking closely at the crossings between the 90th and 91st bands in Fig. 4(b), we find that there is a gap along X-M (R-A), while there are two crossing points along M-Γ (A-Z). These crossing points are parts of the tiny *M*_$z$_-protected nodal rings at the *k*_$z$_ = 0, 2π/*c* planes, which can be easily removed by very small perturbations (without changing the ordering of energy bands at high symmetry *k* points). According to symmetry indicators defined in magnetic systems [32], insulators in type-I MSG 87 are characterized by the indicators $\mathbb {Z}_4 \times \mathbb {Z}_4$ [33,34], which can be explained by two mirror Chern numbers in the *k*_$z$_ = 0 plane. By using the C_4_ eigenvalues [35], we find that the Chern numbers are 0 and 2 for the mirror eigenvalue +*i* and −*i* sectors, respectively, which indicate that nontrivial edge states can emerge on the *M*_$z$_-preserving surfaces.

## CONCLUSION

Based on our first-principle calculations, we find that in the undoped case there are three EPs: the largest EP coming from the Ni $3d_{x^2-y^2}$ orbital, a small EP at Γ from the La $5d_{3z^2-r^2}$ orbital and a relatively larger EP at A from La 5*d*_*xy*_. These Fermi surfaces and symmetry characteristics can be reproduced by our two-band model, which consists of two elementary band representations: *B*_1*g*_@1*a* ⊕ *A*_1*g*_@1*b*. This two-band model could be used for further study of superconductivity in these systems. In the obtained band structure, a band inversion occurred at A, which gives rise to a pair of Dirac points along M-A upon including SOC. The correlation effect of Ni 3*d* orbitals has been estimated in our DFT+Gutzwiller calculation, and the renormalized band structure obtained, which shows that Ni 3*d*_*xy*_ states become very close to *E*_*F*_ around A. A hole pocket is likely induced by hole doping, which may be related to the observed sign change of the Hall coefficient. The potential hole pocket and the band inversion can be captured in the modified model by simply including another Ni 3*d*_*xy*_ orbital. In addition, we show that the nontrivial band topology in the ferromagnetic two-layer compound La_3_Ni_2_O_6_ is associated with Ni $3d_{x^2-y^2}$ and La 5*d*_*xy*_ orbitals.

***Note added*.** While finalizing the present paper, similar works on nickelates were published  [36–40].

## Supplementary Material

nwaa218_Supplemental_File
