# Supplementary material for: Electronic structures and topological properties in nickelates Lnn+1NinO2n+2
Source: Natl Sci Rev. 2020 Sep 2;8(8):nwaa218. doi: 10.1093/nsr/nwaa218 (PMC8363340; doi:10.1093/nsr/nwaa218)
Supplement: nwaa218_Supplemental_File [file nwaa218_supplemental_file.pdf]

SUPPLEMENTARY MATERIAL

A. The band structures of different compounds LnNiO$_2$ (Ln=La, Pr, Nd)

The band structures are in our DFT calculations with SOC for the LnNiO$_2$ (Ln=La, Pr, Nd) compounds.

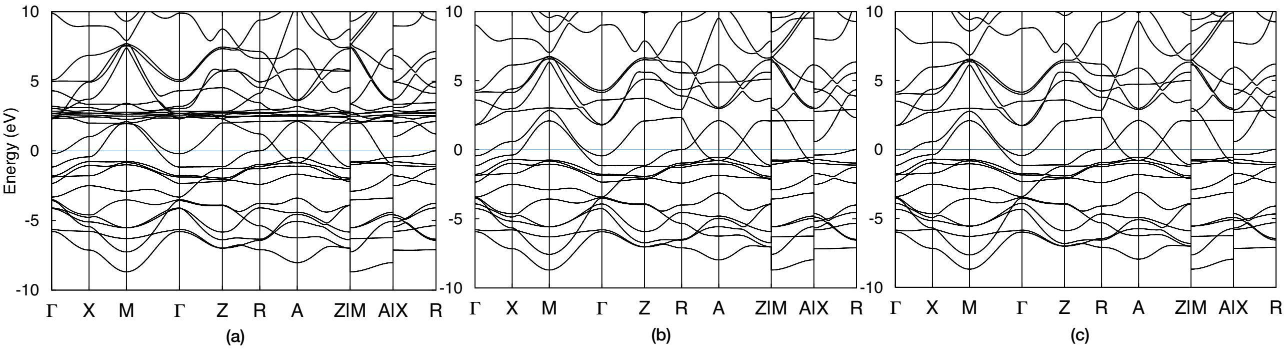

FIG. S1: (Color online) Band structures of LaNiO$_2$ (a), PrNiO$_2$ (b) and NdNiO$_2$ (c). The 4f states of Pr and Nd treated as core states in our PAW potential in our VASP calculations.

B. The evolution of band structures with different U values

The band structure can be affected by the Coulomb interaction, which can be simulated by using the LDA+U method as implemented in VASP. Here we add the on-site interaction on Ni-3d orbitals from U = 1eV to U = 6eV in the LaNiO$_2$ compound. The interaction does not change the overall band structure very much. But, the relative energy difference between the bands of four 3d orbitals (except $d_{x^2-y^2}$) and the other bands ($i.e.$, $3d_{x^2-y^2}$, $5d_{xy}$) increases monotonically as increasing U. As shown in Fig. S2, the band inversion can be removed when U = 6eV, resulting the disappearance of Dirac points.

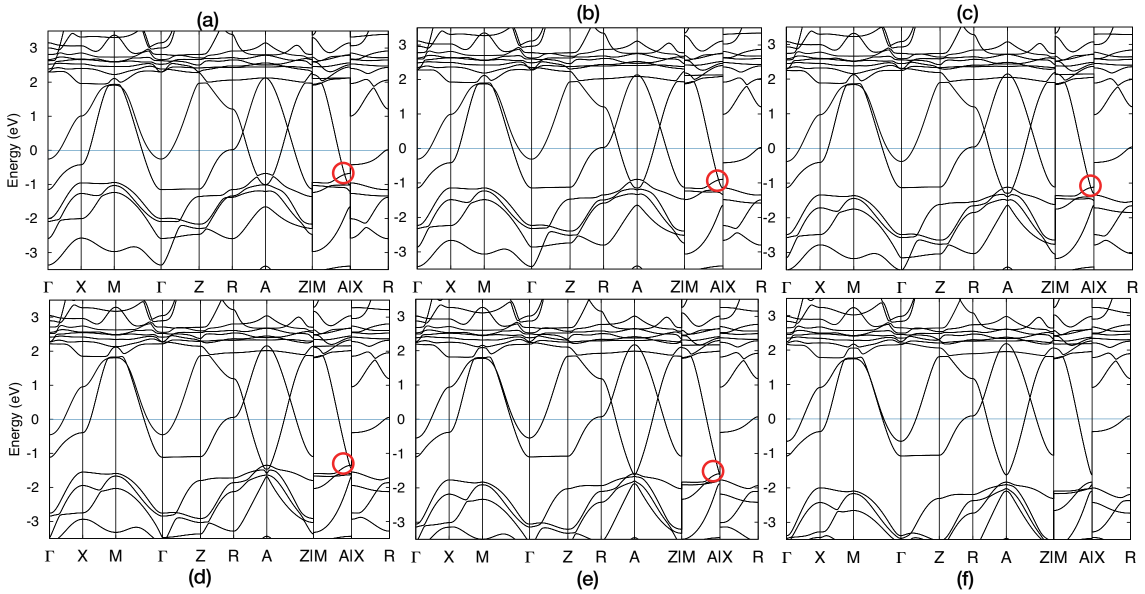

FIG. S2: (Color online) Band structure of LaNiO$_2$ under different Coulomb interactions, from U = 1eV (a) to U = 6eV (f). The Dirac point concerned are indicated by red circles.

C. Irreducible represention for DFT bands with SOC

The irreps of the energy bands with SOC at maximal high-symmetry points are give in Table S1. By using the opensource codes of topological quantum chemistry, the results indicate that it's a Dirac semimetal with 8 occupied bands.

TABLE S1: The irreducible representations (irreps) for the eight occupied bands (4 doubly-degenerate ones) with SOC at the maximal high-symmetry points in SG 123. The irreps are given in ascending energy order. The notation of $Zm(n)$ implies the irrep m at the Z point with the degeneracy of n.

A	Γ	M	R	X	Z
A9(2)	GM7(2)	M7(2)	Z6(2)	R5(2)	X5(2)
A7(2)	GM6(2)	M6(2)	Z7(2)	R5(2)	X5(2)
A7(2)	GM7(2)	M7(2)	Z6(2)	R5(2)	X5(2)
A6(2)	GM6(2)	M6(2)	Z7(2)	R5(2)	X5(2)

D. The four-band model

Here we construct a four band TB model to capture the band topology and Fermi surface structure by using the real atoms' orbitals, in case anyone needs the real atomic orbitals to determine the interacting strength. Based on the analysis of orbital components, we choose our orbitals as: La-d_{xy}, La-d_{z^2}, Ni-$d_{x^2-y^2}$, Ni-d_{xy}, which are referred to as $\alpha = 1, 2, 3, 4$, respectively. The four-band model reads as:

$$H_4(k) = \begin{pmatrix} H_{11} & & & \dagger \\ H_{21} & H_{22} & & \\ H_{31} & H_{32} & H_{33} & \\ H_{41} & H_{42} & H_{43} & H_{44} \end{pmatrix} \tag{1}$$

The space group $\mathcal{G}$ is $P4/mmm$, generated by discrete translation symmetry, inversion, C_{4z}, C_{2x} and C_{2y}.

$$t_{\beta\alpha}^{(l,m,n)} \equiv \langle \beta; 000|\hat{H}|\alpha; lmn\rangle \tag{2}$$

The diagonal terms have the same form as Eq (1) in the main text

$$\begin{aligned} H_{\alpha\alpha} = {} & t_{\alpha\alpha}^{(0,0,0)} + 2t_{\alpha\alpha}^{(1,0,0)}(\cos(k_x) + \cos(k_y)) \\ & + 2t_{\alpha\alpha}^{(0,0,1)}\cos(k_z) + 4t_{\alpha\alpha}^{(1,1,0)}\cos(k_x)\cos(k_y) \\ & + 4t_{\alpha\alpha}^{(1,0,1)}\cos(k_z)(\cos(k_x) + \cos(k_y)) \\ & + 8t_{\alpha\alpha}^{(1,1,1)}\cos(k_x)\cos(k_y)\cos(k_z) \end{aligned} \tag{3}$$

where $t_{\beta\alpha}^{(l,m,n)}$ stands for the hopping amplitude from orbital β of the original cell to α of the (l, m, n) cell as shown in Fig. S3(a):

The anti-diagonal terms are listed as follows

$$H_{21} = 2t_{21}^{(1,1,0)}(\cos(k_x + k_y) - \cos(k_x - k_y)) \tag{4}$$

$$H_{31} = t_{31}^{(1,0,0)}(1 + e^{ik_z})[(1 - e^{ik_y})(e^{-ik_x} - e^{2ik_x}) + (1 - e^{ik_x})(e^{2ik_y} - e^{-ik_y})] \tag{5}$$

$$H_{32} = t_{32}^{(1,0,0)}(1 + e^{ik_z})[(1 + e^{ik_y})(e^{2ik_x} + e^{-ik_x}) - (1 + e^{ik_x})(e^{2ik_y} + e^{-ik_y})] \tag{6}$$

$$H_{41} = t_{41}^{(0,0,0)}(1 + e^{ik_z})(1 + e^{ik_x})(1 + e^{ik_y}) \tag{7}$$

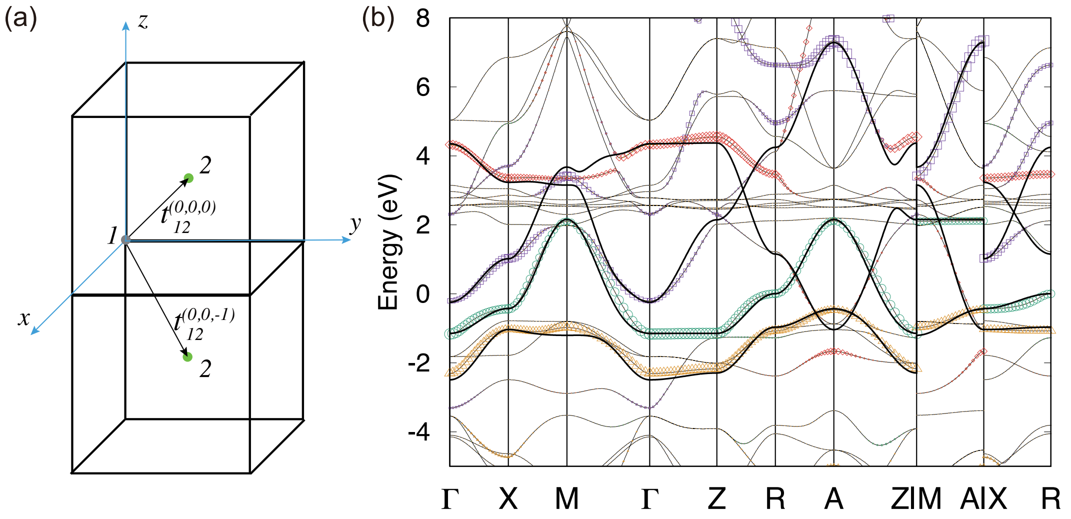

FIG. S3: (Color online) (a) Hopping between different orbitals (b) Band structure of four band tight binding model (black solid line).

TABLE S2: The hopping parameters for the four-band Hamiltonian

$t_{11}^{(0,0,0)}$	2.4319	$t_{22}^{(0,0,0)}$	2.8954	$t_{33}^{(0,0,0)}$	0.1470	$t_{44}^{(0,0,0)}$	-1.2546	$t_{21}^{(1,1,0)}$	0.2720
$t_{11}^{(1,0,0)}$	0.4031	$t_{22}^{(1,0,0)}$	-0.5519	$t_{33}^{(1,0,0)}$	-0.4125	$t_{44}^{(1,0,0)}$	-0.2013	$t_{31}^{(1,0,0)}$	-0.0281
$t_{11}^{(0,0,1)}$	0.5106	$t_{22}^{(0,0,1)}$	-0.7931	$t_{33}^{(0,0,1)}$	-0.0536	$t_{44}^{(0,0,1)}$	-0.0450	$t_{32}^{(1,0,0)}$	0.0281
$t_{11}^{(1,1,0)}$	0.0597	$t_{22}^{(1,1,0)}$	0.0659	$t_{33}^{(1,1,0)}$	0.0894	$t_{44}^{(1,1,0)}$	-0.0638	$t_{41}^{(0,0,0)}$	-0.1286
$t_{11}^{(1,0,1)}$	-0.1366	$t_{22}^{(1,0,1)}$	0.0453	$t_{33}^{(1,0,1)}$	0.0000	$t_{44}^{(1,0,1)}$	0.0156	$t_{42}^{(0,0,0)}$	0.1366
$t_{11}^{(1,1,1)}$	-0.0023	$t_{22}^{(1,1,1)}$	0.0036	$t_{33}^{(1,1,1)}$	0.0134	$t_{44}^{(1,1,1)}$	-0.0069	$t_{43}^{(1,1,1)}$	0.0001

$$H_{42} = t_{42}^{(0,0,0)}(1 + e^{ik_z})(1 - e^{ik_x})(1 - e^{ik_y}) \tag{8}$$

$$H_{43} = 8t_{43}^{(1,1,1)} \cos(k_x)\cos(k_y)\cos(k_z) \tag{9}$$

Using the parameters listed in Table. S2, the band structure of the four band model are shown in Fig. S3(b).
